# Response to Modified Antitubercular Drug Regime and Antiretroviral Therapy in a Case of HIV Infection with Disseminated Tuberculosis with Isoniazid Induced Toxic Epidermal Necrolysis

**DOI:** 10.1155/2012/626709

**Published:** 2012-12-05

**Authors:** Abhijit Swami, Bhaskar Gupta, Prithwiraj Bhattacharjee

**Affiliations:** ^1^Department of Medicine, Silchar Medical College, Silchar 788015, Assam, India; ^2^Silchar Medical College, Silchar 788015, Assam, India

## Abstract

Toxic epidermal necrolysis (TEN) is a potentially life-threatening disorder characterized by widespread erythema, necrosis, and bullous detachment of the epidermis and mucous membranes. Without proper management,TEN can cause sepsis leading to death of the patient. Though TEN is commonly drug induced, Isoniazid (INH) has been uncommonly associated with TEN. As INH is one of the first line drugs in treatment of tuberculosis, TEN induced INH needs modification of antitubercular therapy (ATT) with withdrawal of INH from the treatment regime along with other supportive treatments. Patients with HIV infection and disseminated tuberculosis need to be urgently initiated on an effective ATT on diagnosis of tuberculosis. However, if the patient develops potential life-threatening toxicity to first line antitubercular drugs like INH, an alternative effective ATT combination needs to be started as soon as the condition of the patient stabilizes as most of these patients present in advanced stage of HIV infection and this is to be followed by antiretroviral therapy (ART) as per guidelines. The present case reports the effectiveness of an ATT regime comprising Rifampicin, Pyrazinamide, Ethambutol, and Levofloxacin along with ART in situations where INH cannot be given in disseminated tuberculosis in HIV patients.

## 1. Introduction

 Tuberculosis (TB) in all forms has been associated with all stages of HIV infection. TB is one of the most common coinfections in HIV patients across all continents especially at low CD4 counts [1, 2]. By producing a progressive decline in cell-mediated immunity, HIV alters the pathogenesis of tuberculosis, in coinfected individuals and leading to a more disseminated disease [3]. WHO recommends testing for HIV in newly diagnosed TB patients [4].

 HIV patients with TB coinfection are in Stages III or IV depending upon the site of organ involvement [5]. As per the latest international and Indian guidelines, HIV infected patients with TB co-infection need to be initiated with Rifampicin, Isoniazid, Ethambutol, and Pyrazinamide based antitubercular therapy (ATT) urgently in standard dosages followed by antiretroviral therapy (ART) within 2–4 weeks [6, 7] of starting ATT [8].

 Drugs used for ATT have their own adverse effects and some of them can be life threatening and need intensive therapy once they develop [9]. HIV infected patients are more prone to the adverse effects of ATT than non-HIV patients [10]. The situation becomes even more serious if the adverse effects of ATT develop in a patient in advanced stages of HIV infection as ATT has to be started in these patients before initiation of ART.

 We present a case of newly diagnosed Stage IV HIV infection with disseminated tuberculosis that developed toxic epidermal necrolysis when ATT was initially started with standard regime and the gratifying response to alternate antitubercular drugs.

## 2. Case History

Mrs. ID, a 43-year widow, was admitted into a private hospital with history of chronic diarrhea, low grade fever, and weight loss of two-month duration. After hospitalization, the woman had an ultrasound examination of abdomen which revealed abdominal lymphadenopathy. On the basis of ultrasound findings, she was diagnosed to be a case of tuberculous lymphadenopathy. Following the diagnosis, Antitubercular drugs (Rifampicin, Isoniazid, Ethambutol, and Pyrazinamide) were started in a standard dosage. However, she started developing oral and mucosal ulcerations from the 6th day of antitubercular therapy (ATT) and by day 12 of the ATT. She had developed extensive skin involvement along with burning sensation during micturition and cough with expectoration for which she was referred to Silchar Medical College for further treatment.

 On admission into our hospital, the woman was found to be dehydrated and cachetic with body weight of 40 kg and BMI of 19.2 kg/sq m. She had mild pallor with bilateral cervical and axillary lymphadenopathy. Her vital signs were stable. She was afebrile, conscious with Glasgow Coma Scale of 15/15. She had irregularly shaped erythematous macules with central purpuric areas over palms and soles along with large areas of erosions over chest and proximal limbs. On her back, detachment of full thickness of epidermis was noted with dark oozing fluid from the denuded areas. Over the trunk large flaccid bullae were present with positive Nikolsky's sign and as a whole more than 40% of body surface area was affected. The scalp was not affected.

 The woman also had hemorrhagic cheilitis, stomatitis with increased salivation, conjunctival congestion, photophobia, and increased lacrimation. There were areas of erosions over labia majora and mucosa. Few crepitations were found on her right lung during auscultation of her respiratory system. She did not have any abdominal organomegaly.

 Clinically, she was diagnosed to be a case of disseminated tuberculosis with drug induced toxic epidermal necrolysis (TEN) along with pneumonia of right lung. Possibility of associated bacterial pneumonia was considered as she developed cough with expectoration after hospitalization and almost a week after initiation of anti-tubercular therapy.

 Laboratory investigations revealed hemoglobin level of 9 gm/dL, total leukocyte count of 9700/cu·mm, differential count of PMN 75% lymphocytes 18%, eosinophil 6%, and basophils 1%. Her ESR was 72 mm AEFH. She was euglycemic with normal renal functions and electrolyte levels. Her alkaline phosphatase was 313 U/L. After hospitalization, she was tested for HIV with her consent and she tested positive for HIV-1. Her chest X-ray showed nonhomogenous opacities in her right lung lower lobe. Sputum tested negative for TB bacillus. Urine analysis was normal. Repeat ultrasound examination of abdomen revealed multiple mesenteric lymph glands with the biggest measuring 2 cm in diameter. There was growth of *Staphylococcus aureus* from culture of her skin lesions sensitive to Vancomycin, Levofloxacin, and Linezolid.

 Needle aspiration from axillary and cervical glands showed acid fast bacilli on ZN stain. Her CD4 count was 57/*μ*L. She tested negative for hepatitis B, C and syphilis.

 Her antitubercular drugs were stopped immediately on admission and the patient was kept in isolation. Her skin lesions were covered with nonadherent protective dressings containing petroleum gauze. She was given Ceftriaxone 1 gm IV 12 hourly combined with Levofloxacin (L) 500 mg per day for pneumonia. She was also given tab Cetrizine to relieve the pruritus. As the lady was able to take oral and liquid foods orally, she was encouraged to take food orally. Her vital signs and fluid and electrolyte balance were closely monitored.

 After stoppage of her ATT, the patient did not have any extension of her skin and mucosal lesions. Her skin lesions started to heal by the 6th day of hospitalization and within two weeks of admission her skin and mucosal lesions had almost healed. After improvement of her skin lesions, Cotrimoxazole (double strength) was started with close monitoring. INH was started at 50 mg once daily but she immediately developed recurrence of her skin lesions and INH had to be stopped. Rifampicin (R) was then started at a gradually escalating dosage till she could tolerate 450 mg Rifampicin once daily given before breakfast. Her skin and mucosal lesions along with her liver enzymes were monitored regularly. Tab Ethambutol (E) and Pyrazinamide (Z) were subsequently introduced without any recurrence of her cutaneous problems. After 15 days of ATT with 4 drugs (R + E + Z + L) and with improvement of her physical conditions, ART was started with Stavudine, 30 mg twice daily, Lamivudine 150 mg twice daily, and Efavirenz–600 mg (S/L/E) daily.

 The woman improved steadily and was discharged with R + Z + L + E, Cotrimoxazole, and ART. After 6 months of therapy with anttubercular and antiretroviral drugs, her CD4 was 208/*μ*L and Hb was 10.7 g/dL with normal ALT. She weighed 43 kg after 6 months. She continues to be on S/L/E, Cotrimoxazole prophylaxis, and ATT with plan to stop ATT at 9 months.

## 3. Discussion

 Toxic Epidermal Necrolysis (TEN) was first described by Lyell in 1956 [11]. It is a potentially life-threatening dermatologic disorder characterized by widespread erythema, necrosis, and bullous detachment of the epidermis and mucous membranes, resulting in exfoliation and possible sepsis and/or death. Mucous membrane involvement can result in gastrointestinal hemorrhage, respiratory failure, ocular abnormalities, and genitourinary complications. TEN is most commonly drug induced. However, the disorder has other potential etiologies, including infection, malignancy, and vaccinations. TEN is idiosyncratic, and its occurrence is not easily predicted [12].

TEN following INH has been reported infrequently in the literature [13]. INH remains a vital component of antitubercular therapy. Any HIV patient coinfected with tuberculosis needs to be put on ATT immediately. If the patient develops adverse effects on any first line antitubercular drugs, the treatment of tuberculosis needs to be modified. The mortality rate is increased if an HIV patient develops TEN [14].

 TEN has been reported with INH/Rifampicin combination during initiation of ATT in HIV positive individuals and has been fatal in many cases [15]. Treatment of TEN involves removing of the offending agent followed by monitoring of fluids and electrolytes, proper wound care, nutritional support, and supportive systemic therapy including broad spectrum antibiotics though the use of prophylactic antibiotics is debatable [16].

 ATT is to be restarted in ATT induced TEN after the reaction resolves according to a set protocol [17] to identify the culprit drug. In the present case, INH was the drug responsible as TEN developed every time INH was started and TEN resolved after discontinuation of INH. In case of INH intolerance ATT regime needs to be modified and is to be continued with Rifampicin, Ethambutol, and Pyrazinamide for 6–9 months [18]. As per WHO guidelines, Efavirenz is the preferred Nonnucleoside reverse transcriptase inhibitor (NNRTI) in the NNRTI based ART regime in patients with TB coinfection on Rifampicin based ATT [19].

 Response to ART in HIV patients can be assessed by changes in CD4 count from initial values at periodic intervals after the beginning of ART. An adequate CD4 response for most patients on therapy is defined as an increase in CD4 count in the range of 50–150 cells/mm per year, generally with an accelerated response in the first 3 months [20].

 The case is interesting because the lady presented in Stage IV HIV infection with disseminated tuberculosis and developed INH induced TEN. There was an urgent need for ATT in this patient. As INH is one of the most potent antitubercular drugs available, alternate ATT had to be started as per standard protocol with close monitoring of the patient along with judicious supportive therapy. Subsequent introduction of ART helped to improve her condition both clinically and immunologically when she was reassessed at six months of antiretroviral therapy.
